# Pharmacological Activities of Schiff Bases and Their Derivatives with Low and High Molecular Phosphonates

**DOI:** 10.3390/ph16070938

**Published:** 2023-06-28

**Authors:** Ivelina Tsacheva, Zornica Todorova, Denitsa Momekova, Georgi Momekov, Neli Koseva

**Affiliations:** 1 Institute of Polymers, Bulgarian Academy of Sciences, Acad. G. Bonchev Street, 1113 Sofia, Bulgaria; 2 Department of Pharmaceutical Technology and Biopharmaceutics, Faculty of Pharmacy, Medical University of Sofia, 1000 Sofia, Bulgaria; 3 Department of Pharmacology, Pharmacotherapy and Toxicology, Faculty of Pharmacy, Medical University of Sofia, 1000 Sofia, Bulgaria; 4 Bulgarian Academy of Sciences, 1 “15 Noemvri” Str., 1040 Sofia, Bulgaria

**Keywords:** anthracene-containing schiff bases, furan-containing Schiff bases, aminophophonates, poly(aminophosphonate)s, pharmacological activities

## Abstract

This review paper is focused on the design of anthracene and furan-containing Schiff bases and their advanced properties as ligands in complex transition metal ions The paper also provides a brief overview on a variety of biological applications, namely, potent candidates with antibacterial and antifungal activity, antioxidant and chemosensing properties. These advantageous properties are enhanced upon metal complexing. The subject of the review has been extended with a brief discussion on reactivity of Schiff bases with hydrogen phosphonates and the preparation of low and high molecular phosphonates, as well as their application as pharmacological agents. This work will be of interest for scientists seeking new challenges in discovering advanced pharmacological active molecules gaining inspiration from the versatile families of imines and aminophosphonates.

## 1. Introduction

**Scheme 1:**
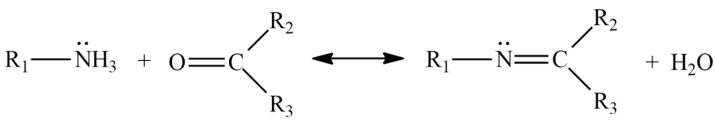
General scheme of Schiff base formation [1].

Schiff bases present an important class of compounds that contain the azomethine (–C=N–) group in their structure. They are formed by the reaction between a primary amine and an aldehyde or a ketone under specific conditions. Schiff bases belong to the versatile family of imines. The general structure of Schiff bases and the synthetic path to their formation are presented in Scheme 1. The first imines were prepared in 1864 by Hugo Schiff via a condensation reaction between a carbonyl compound and an amine [1].

Though other ways of obtaining Schiff bases have been invented, the reactions between amines and either aldehydes or ketones present the most exploited method. While the aldehydes react with primary amines upon mixing to form Schiff bases, the reaction with ketones depends on the type of catalyst, pH range, the type of solvent and the reaction temperature [2].

**Scheme 2:**
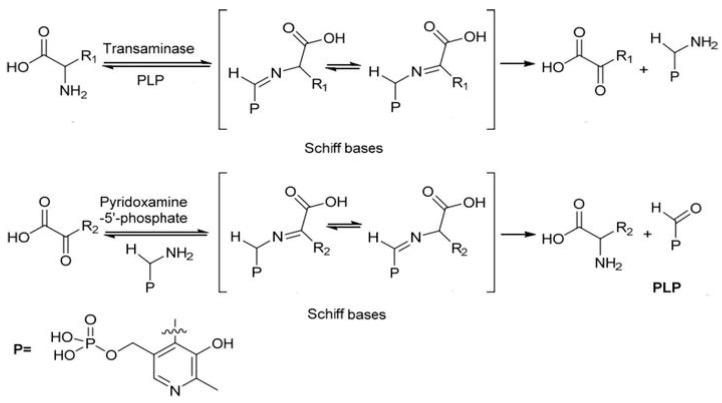
Transamination reaction [2].

One of the most important properties of Schiff bases is their selectivity toward metal ions and ability to form complexes, including highly stable 4-, 5- and 6-ring complexes. Just like the Schiff bases, their metal complexes also possess anticancer, antibacterial, antifungal and antiviral properties [2,3,4,5,6,7,8,9,10]. In biology, the Schiff bases play a very important role as a transport agent in amino acid biosynthesis (Scheme 2) [2].

For the last decades, a lot of research groups have been investigating Schiff bases and reporting on the huge scope of bioactivities these compounds possess–antiviral [11,12], antifungal [13], antibacterial [13,14], antioxidant [14,15], anti-inflammatory [15], antitumor [16,17], anticancer [18] and antimicrobial properties [17,18,19]. Additionally, their potential use as urease inhibitors has also been suggested [11,12,13,14,15,16,17,18,19]. The interest in Schiff bases has been rising with time due to the fact that they can be synthesized with sufficient purity and high yield. Furthermore, the presence of donor groups, such as N, O, S, and P, ensures their ability to form stable complexes with almost all metals. They can also be functionalized with many different compounds. Schiff bases can be precursors for the obtaining polyamine derivatives, which can be further modified due to the presence of NH-groups in their structure.

The variety of structures and properties broadens the application of Schiff bases covering chemical catalysis and chemical analysis, diagnosis and therapy, new technologies such as television and computer screens, digital clock displays, etc. [20,21,22,23,24,25]. Additionally, Schiff bases play roles in biological systems and as intermediate in enzymatic reactions [26].

The combination of a carbon nitrogen double bond with heteroatom rings leads to the enhancement of most of the above-mentioned abilities of the Schiff bases [27]. Such compounds are the furan- and anthracene containing Schiff base derivatives, which can be very important in pharmaceutical and biological science, due to their intensified biological activity. The presence of furanyl and anthracenyl moieties augments the level of conjugation in the molecular systems, imparts antioxidant activity and influences their physico-chemical characteristics such as solubility, metal-complexing, spectral properties, etc. The incorporation of such fragments into the molecular structure brings benefits that have motivated the research on the molecular design of furan and anthracene derivatives which are broadly discussed in the following sections of the review paper.

Increasing knowledge of cancer chemotherapy, it is needful to design new compounds to overcome the resistance of available anticancer drugs. α-Aminophosphonates are an important class of compounds that have received wide attention due to various pharmaceutical applications such as anticancer, antiviral, antibacterial drugs, antibiotics, etc. [28,29,30]. Aminophosphonates are organophosphorus compounds structural analogues of α-aminocarboxylic acids, in which instead of the planar carboxylic group the compound possesses a phosphoric acid residue with a tetrahedral geometry [31]. They consist of P–C–N moiety, which have an effect on their potential biological activity [32]. They showed a low inhibition of cell vitality of mammalian cells [33] which defines them as promising drug candidates [34,35] and necessitates the extensive study of their pharmacological potential [36].

**Scheme 3:**
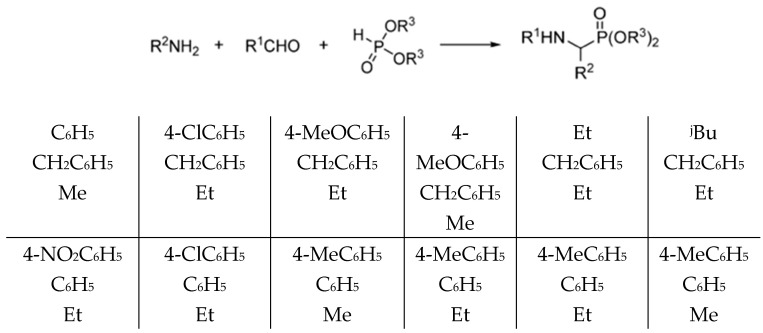
General scheme of Kabachnik–Fields reaction [30].

Three outstanding scientists Kabachnik, Medved and Fields discovered in 1952 independently the reaction known nowadays as the Kabachnik–Fields reaction [37] following the classical methods for synthesizing organophosphorus compounds. Kabachnik–Fields reaction is the most commonly used reaction to acquire aminophosphonates and poly(aminophosphonate)s. The reaction proceeds in a three-component system: a hydro phosphoryl compound, a carbonyl compound (aldehyde or ketone) and an amine, resulting in α-aminoalkylphosphonates commonly named as α-aminophosphonates (Scheme 3) [38,39,40,41,42,43,44,45].

**Scheme 4:**
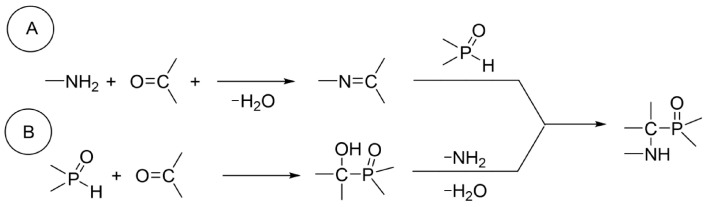
General mechanism of Pudovik reaction [30].

Another known reaction for C–P bond formation is the Pudovik reaction, which is often named as the phospha-Michael addition (Scheme 4). It is one of the most feasible and fundamental methods for generating C–P bond. The reaction is based on the addition of P(O)–H bonds to electron-deficient alkenes in the presence of an alkali catalyst, normally the alkaline alkoxides in alcoholic solutions. The reaction has found a broad range of applications in organic synthesis and in industrial chemistry because of its atoms’ economic nature and the commercial availability of the starting materials [39,40,41,42,43,44,45,46,47,48].

On the basis of our scientific experience in organic and polymer synthesis in collaboration with colleagues from biological and pharmaceutical sciences, the idea arose to combine in a joint review paper results of research that cover the synthesis of biologically active Schiff bases and their transformation into low- and high-molecular aminophosphonates with determination and analysis of their biological activity. To the best of our knowledge, such summary material has not been published, and we believe that it will be useful both for those studying and working in the field of organic, polymer and medicinal chemistry, as well as in the design of drugs, macromolecular biodegradable compounds with intrinsic bioactivity, development of therapeutic and diagnostic systems.

## 2. Furan-Containing Schiff Bases

**Scheme 5:**
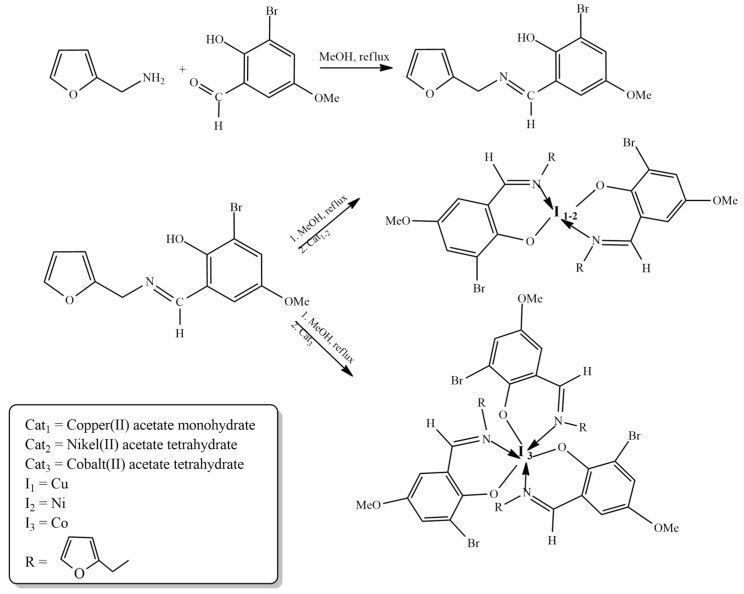
Synthesis of a furan-containing Schiff base, and its complexing with transition meals [49].

As mentioned above, Schiff bases comprising heterocyclic moieties are gaining a lot of interest due to their ability to act as ligands and to bind multiple biological molecules. An example of such a heterocyclic compound is furan—a 5-membered ring containing four carbons and one oxygen. Furan-based offshoots, such as nitrofurantoin, furazolidone, nifuratel and nifurtimox are frequently used in drug research due to the fact that the presence of furanyl moiety in conjugated systems enhances the level of conjugation and improves their solubility and the transport properties. The use of furan-alternatives in bioorganic chemistry is considered very promising due to their ability to mimic part of the structure of some natural and pharmacological compounds. Vankateswarlu et al. reported the synthesis of a large number of transition metal complexes of various biologically active Schiff bases containing furan as core unit. They demonstrated the obtaining of Cu (II), Ni (II) and Co (III) complexes (Scheme 5). It was proved that the complexes exhibit good antioxidant activity and better antimicrobial activity towards *Bacillus thuringiensis*, *Streptococcus pneumoniae*, *Escherichia coli* and *Pseudomonas putida* compared to the free ligand [49].

**Scheme 6:**
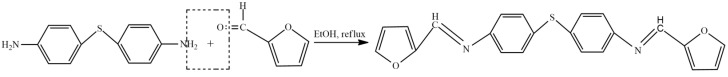
Synthesis of 4,4-bis(2-furane carboxaldehyde)diphenyl diimino sulfide (X=O) [50].

Nowadays, there is an urgent demand for novel antimicrobial agents for a wide application of fields such as hygienic application, hospital, dental surgery equipment, medical devices, health care, water purification system, textiles, storage and food packaging. Mesbah et al. present the synthesis of three new Schiff bases derived from condensation of aldehydes with 4,4-diaminodiphenyl sulfide. In their work, one of these aldehydes is a furan-based compound-furan-2-carboxaldehyde (Scheme 6). The obtained 4,4-bis(2-furane carboxaldehyde)diphenyl diimino sulfide was proven to show an antimicrobial effect against *Escherichia coli* and strong antimycotic activity against *Microsporum canis* [50].

**Figure 1:**
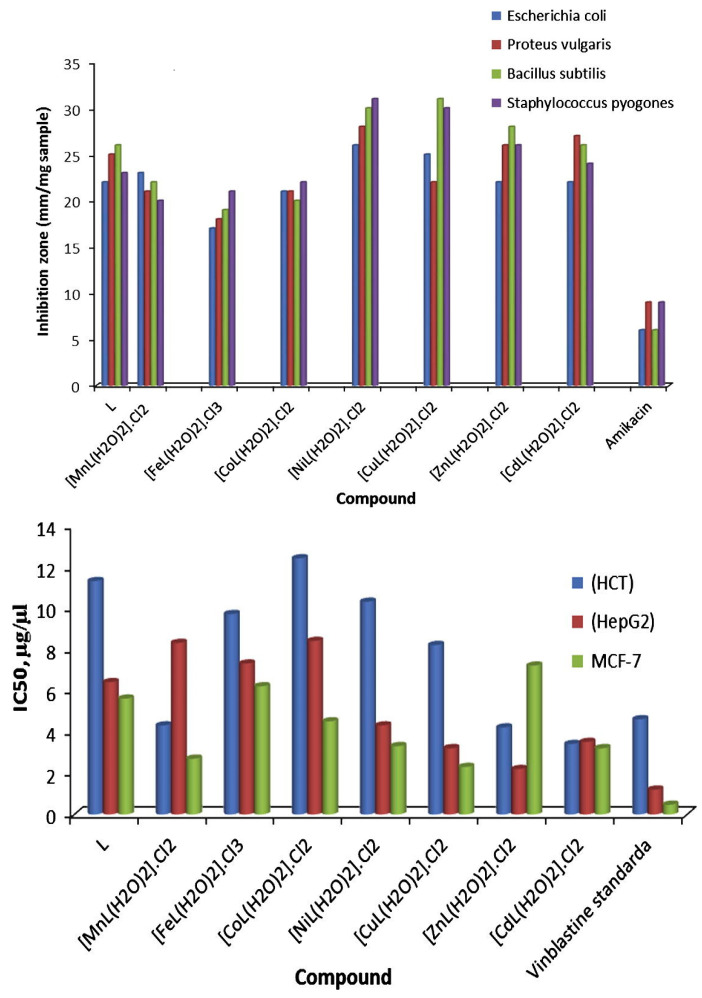
Antibacterial activity and cytotoxicity against cancer cell lines of Schiff base complexes [51].

Mohamed et al. prepared a novel furan-based diimine [N1,N3-bis(furan-2-ylmethylene)propane-1,3-diamine] via a condensation reaction between furan-2-carboxaldehyde and propane-1,3-diamine. The novel bis-Schiff base was used as ligand in the complex formation with a number of transition metal ions (Co(II), Ni(II), Cu(II), Mn(II), Cd(II), Zn(II) and Fe(III)) which demonstrated higher antimicrobial effect than the free bis-Schiff base as shown in Figure 1. In addition, Co(II), Cu(II) and Zn(II) complexes exhibited significantly higher antibacterial activity than the standard antibiotic amikacin drug (Figure 1) [51]. The cytotoxicity evaluation was carried out on a panel of human malignant cell lines, namely: MCF-7, HepG2 and HCT. The majority of the complexes showed higher activity towards the human colorectal carcinoma cell line HCT as compared to other two treated cell lines [51].

**Scheme 7:**
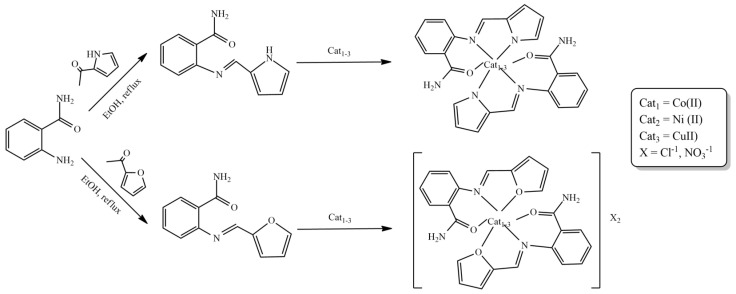
Preparation of Schiff base ligands obtained from 2-aminobenzamide and their metal complexes [52].

According to the literature, not much scientific works have been reported on the preparation of complexes of transition metals with Schiff base derivatives of 2-aminobenzamide providing oxygen and nitrogen donors. Therefore, Tyagi et al. presented in their research the synthesis of a Schiff base derived from the reaction of 2-aminobenzamide with furan-2-carboxaldehyde and their complexes with Ni(II), Cu(II) and Co(II) metal complexes (Scheme 7). They were thoroughly characterized by means of different spectroscopic techniques, thermal methods, DFT studies and antimicrobial tests. The authors proved that the newly synthesized Schiff base ligands act as tridentate ligands. Their metal complexes possess higher antimicrobial activity against different bacterial and fungal strains most probably due to azomethine linkage, while also possessing increased activity upon coordination with different metal ions. Thus, the metal complexes could be regarded as candidates for the development of potent antimicrobial drugs [52].

A Schiff base (Z)-4-((furan-2-ylmethylene)amino)benzenesulfonamide was synthesized via a reaction between furfural and sulfanilamide. The new compound exhibited a moderate antimicrobial activity compared to a standard drug. A molecular simulation was performed on the compound attached to a human mitochondrial (2WYA) protein and an excellent binding affinity score was observed [53].

**Scheme 8:**
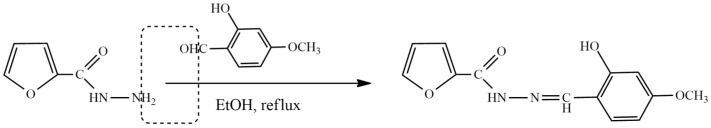
Obtaining of Schiff base ligand for Al^3+^ detection [54].

Another interesting application of furan-containing Schiff bases was reported by Peng et al. [54]. They seized the need to elaborate a reliable technology for fast and selective identification of Al^3+^ in ecological and biological systems. Since aluminum is one of the most abundant elements on the planet, it is extensively used by many industries, thus polluting the environment and harming the human body. Fluorescent detection technique has been often used for the detection of Al^3+^, but it has also some limitations, such as the complicated synthetic procedures for the preparation of the samples, long response time and poor selectivity. To overcome these limitations Peng et al. prepared a Schiff base derived from furan- 2-carbohydrazide and 2-hydroxy-4-methoxybenzaldehyde (Scheme 8). The new molecule presented an efficient Al^3+^ fluorescent probe which exhibited excellent selectivity and sensitivity towards Al^3+^ [54].

Similar studies aimed to design an effective and reversible Al^3+^ fluorescent chemosensor applicable in aqueous media. Therefore, a PEG chain was anchored to a furan Schiff base to yield a functionalized PEG (PEGFB). The product exhibits excellent selectivity and sensitivity to Al^3+^ with turn-on fluorescence in a water environment. Furthermore, the detection limit of the ions was very low and a practical application was found for PEGFB in preparing test strips coated with PEGFB to be used in aqueous environment [55].

**Scheme 9:**
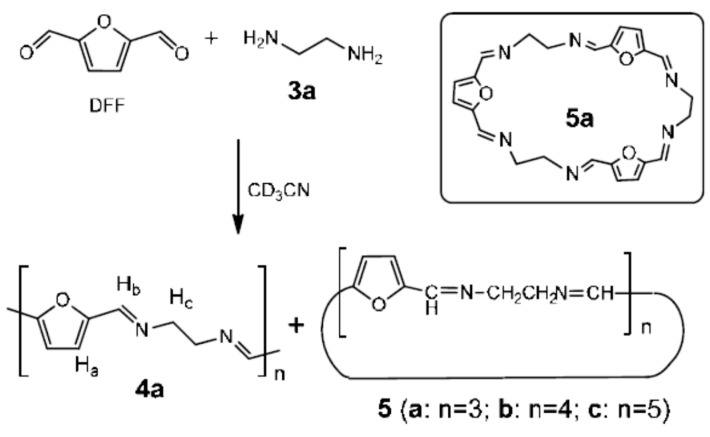
Products obtained via the reaction of 2,5-diformylfuran and 1,2-diaminoethane [56].

Aside from the low-molecular weight Schiff bases, the polymeric Schiff bases are also advantageous materials, possessing all of the characteristics of the monomeric Schiff bases, but expanding them due to the macromolecular character of the compound. Polymeric Schiff bases (or polyimines) are a class of macromolecular compounds possessing the –CH=N− repeating structural unit. They exhibit adequate thermal stability and useful mechanical properties. Aromatic polyimines find application as high-strength fibres, while their metal complexes are used as catalysts and dyes. Unfortunately, most of the polymeric Schiff bases are not soluble due to strong chain–chain interactions arising from the polar -C=N- groups. On the other hand, furan-based polymeric materials possess potential as sustainable materials and can be used for deriving readily available furan-based chemicals from biomass. Unlike benzene, the furan ring exhibits a diene character that could potentially impact the properties of furan-containing materials. Even though one of the most suitable monomers for obtaining polymeric Schiff bases is 2,5-diformylfuran (DFF) due to its two aldehyde groups, only a few examples of polymeric furan analogues have been synthesized from DFF, because of the high cost of the DFF monomer. Xiang et al. expanded the library of furan-containing polymeric Schiff bases by synthesizing two polymeric Schiff bases via polymerization of 2,5-diformylfuran with two primary amines (1,2-diaminoethane, 4,4-diaminobibenzyl) in acetonitrile and ethanol at room temperature. The products of the polycondensation of 2,5-diformylfuran and 1,2-diaminoethane presented cyclic oligomers and linear polymers (Scheme 9). The polymeric products exhibited good thermal stability [56].

Furan is quickly and readily absorbed from the lung and the intestine, can pass through the body diffusional barriers and enters different organs. Thus, molecules containing the furan or tetrahydrofuran ring are pharmacologically active and included in several drugs [57].

These interesting biological properties of furan-containing compounds provoke the interest of researchers, including ours, to explore the possibilities of pharmacological applications of these furan-containing aminophosphonates. Lewkowski et al. tested the antibacterial activity of aminophosphonates containing 2-nitrofuran group and two N-aryl-5-nitro-furfuralaldimines. They found that the biological activity of received aminophosphonate enantiomers can differ greatly. The near concentrations for cytotoxicity and antibacterial efficacy can be improved by resolution and a separate evaluation for the enantiomers [58]. The same research group studied furan-containing dibenzyl and diphenyl aminophosphonates for their antiproliferative potential on two cell lines of colorectal cancer-HCT116 and HT29. They found that the furan-containing aminophosphonates were more anti-proliferative active than the corresponding diphenyl derivatives [59].

Patnala et al. have also investigated methods of obtaining furan-containing α-diaminophosphonates and their pharmaceutical applications. One of the compounds, a furan moiety in its structure, showed in vivo antiviral activity against tobacco mosaic virus (TMV) similar to Ningnanmycin as a standard drug [60]. α-Aminophosphonates bearing a furan ring were screened for potential anti-Alzheimer activity. All compounds showed inhibition against BuChE (IC_50_ = 2.30–18.59 µM) and AChE (IC_50_ = 0.88–4.46 µM) and acceptable antioxidant activity [61].

**Scheme 10:**
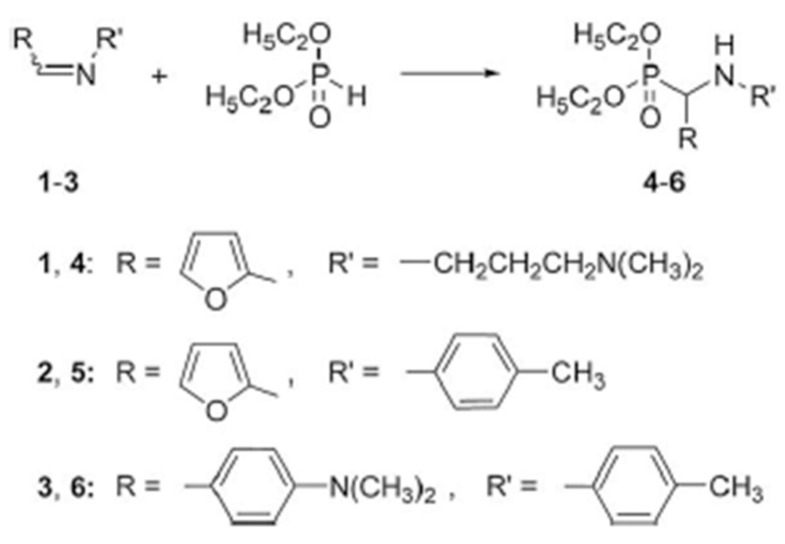
Synthesis of furan-containing α-aminophosphonic acid diesters [62].

In 2009, our research group proposed the design of novel furan-containing α-aminophosphonic acid diesters. Three novel compounds were obtained from the addition reaction of diethyl H-phosphonate to furan- and toluidine-containing Schiff bases: p-[N-methyl(diethoxyphosphonyl)-(2-furyl)toluidine;p-[N-methyl(dietoxyphosphonyl)--(4-dimethylaminophenyl)]toluidine;N,N-dimethyl-[N’-methyl(diethoxyphosphonyl)--(2-furyl)]-1,3-diaminopropane (Scheme 10).

The starting furan-containing Schiff bases and their a-aminophosphonates were evaluated for antiproliferative activity against four human leukemic cell lines, together with the multi-drug-resistant model HL-60/Dox. All compounds exerted concentration-dependent cell growth-inhibition effects after 72 h exposure [62].

It is noteworthy that the furyl and toluidine-containing Schiff base (2) and the corresponding aminophosphonate (5) have similar cytotoxicity to cisplatin for the K-562b cell line (Table 1). Based on classification of the National Cancer Institute of US, the compounds 2 and 5 exhibited considerable to high cytotoxic activity against the K-562^b^ cell line and moderate activity against LAMA-84 and multi-drug-resistant phenotype of the acute promyelocyte leukemia (HL-60/DOX) cell line [63]. Similarly, aminophosphonate 6 displayed varying cytotoxic activity against the studied cell lines. The obtained IC_50_ values presented in Table 1 suggested that the cytotoxic activity of the two aminophosphonates and the Schiff base 2 might be specific to cancer cell lines. These results gave us the motivation for further studies. That is why it supposes the availability of both furyl and N-tolyl units is an essential prerequisite for the acceptable activity in these materials. The investigated materials were far less active as liken to the referent anticancer drug Cisplatin than the aminophosphonate bearing furyl and N-tolyl parts whose effect on K-562 cell line was similar to the control. This aminophosphonate was shown to induce oligonucleosomal DNA fragmentation, which implied that the induction of cell death through apoptosis implements an important role in its cytotoxicity [62]. The capability of these substances to selectively inhibit MRP-1 expressing HL-60/Dox supposes that they could be regarded as encouraging leads for further elaboration of agents active in chemotherapy refractory malignant sickness.

The cytotoxic results give us reason to continue looking for new molecules and further develop furyl and toluidine-containing Schiff bases and the corresponding aminophosphonates and their potential application as pharmacological agents. The received collateral sensitivity of multi-drug-resistant cancer cell lines and the established activity of representative substances that trigger apoptosis at sub-cytotoxic levels offer that these substances can be studied for further pharmacological applications [62].

Ν,Ν-dimethyl-[N’-methyl (diethoxy-phosphonyl)-(2-furyl)]-1,3-diaminopropane as furan-containing aminophosphonate was investigated for in vitro cytotoxic activity on six human epithelial cancer cell lines. Testing for Safety was conducted in vivo on ICR mice and in vitro (3T3 NRU test) for antiproliferative activity and genotoxicity. The aminophosphonate demonstrated high in vitro cytotoxic activity against the 647-V cell line (human bladder carcinoma) and the HepG2 cell line (human hepatocellular carcinoma). This furan-containing aminophosphonate possessed a moderate genotoxic and antiproliferative activity in vivo and presented a complete absence of toxicity to Balb/c 3T3 (clone 31) mouse embryo cells [64].

## 3. Anthracene-Containing Schiff Bases

**Figure 2:**
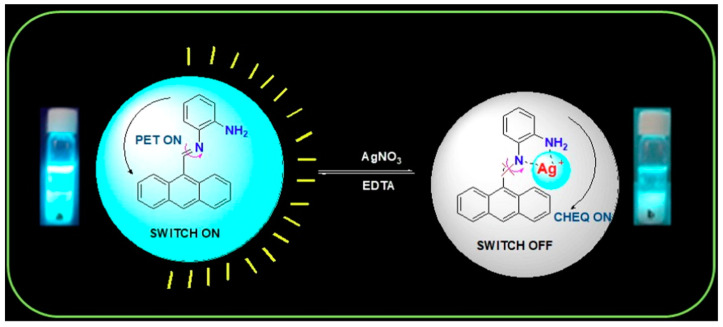
Schiff base-anthracene chemosensor [66].

Chemical ingredients containing anthracene framework possess prolonged π-conjugation property. They are also very stable, give a good quantum yield and demonstrate influential photoluminescence possibilities which lead them to be considered as promising chemosensors [65]. Suguna et al. investigated an anthracene based chemosensor that selectively sensed silver ions by quenching the fluorescence intensity through a “Switch On-off” process. They also investigated the finding of silver ions in water and soil samples as well as the changes in the solid support silica gel color under the UV light with and without silver ions for a potential usage of the agent as a fingerprint developer agent (Figure 2) [66].

Sek et al. investigated the optical and redox properties of anthracene-delivered Schiff bases containing in their structure anthracene, naphthalene, phenyl, biphenyl, triphenlyamine and phenanthrene. Introduction of second anthracene unit resulted in shift of absorption to lower energy region in comparison with other imines [67].

**Scheme 11:**
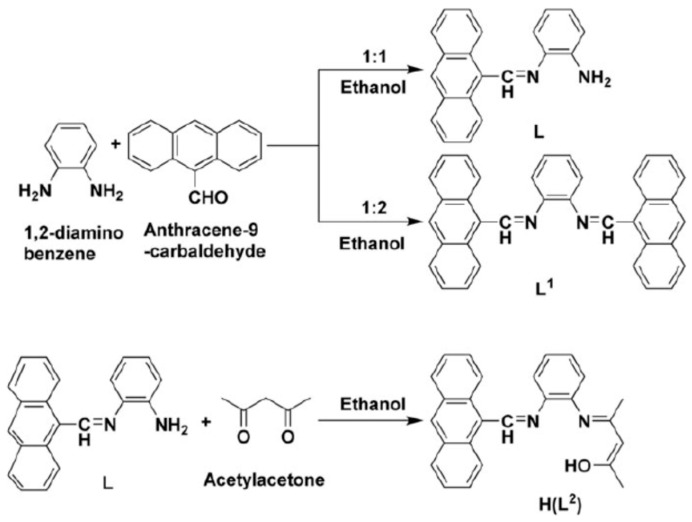
Synthesis of anthracene-based Schiff base ligands [68].

The presence of transition metals in living organisms is of great importance especially for their storage and transport in human blood plasma. Transition metal ions are responsible for bioprocesses such as cell division, respiration, nitrogen fixation and photosynthesis. Schiff base complexes are considered as potential biomimics model compounds. They can form complexes not only with one, but also with several metal ions and thus having similar structure and function to those found in living organisms, like enzymes and proteins [67]. Gubendran et al. report the preparation of new anthracene derived Schiff base ligands and their Cu(II) complexes (Scheme 11). The results of their study revealed a high binding affinity of one of the complexes with DNA via groove mode of interaction and a significant binding ability of another of their complexes through electrostatic interaction with DNA helix. The authors considered the so elaborated Cu(II) complexes as perspective drug candidates [68].

**Scheme 12:**
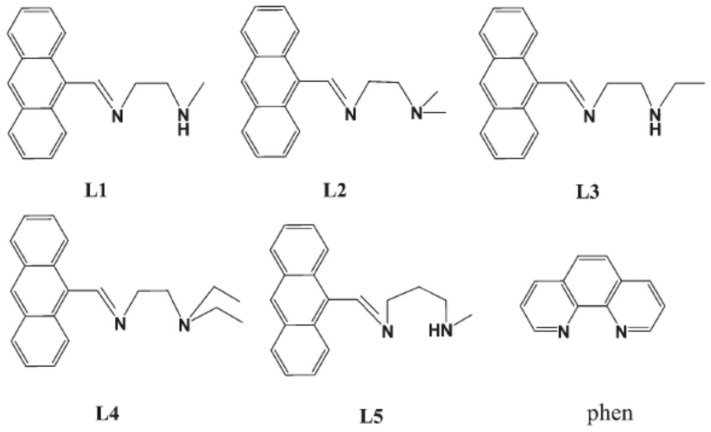
Structures of anthracene-based ligands for Cu(II) complexation [69].

Jaividhya et al. reported that Cu(II) diimine complexes of different primary ligands could bind and cleave DNA, while exhibiting distinguished cytotoxicity such as certain mixed ligands μ-phenoxo-bridged dinuclear Cu(II) complexes with diimine co-ligands (Scheme 12). They also exhibited efficient chemical nuclease and protease activities and cytotoxicity. The authors proved that Cu(II) complexes of phenolate and 3N ligands bind with DNA covalently and were found to be non-toxic to normal cells. In the same work it was also reported that ligand Cu(II) complexes non-covalently binding with DNA displayed higher cell viability inhibition on human HBL-100 breast cancer lines compared to the corresponding 1:1 ligand: metal complexes and referent drug [69].

**Scheme 13:**
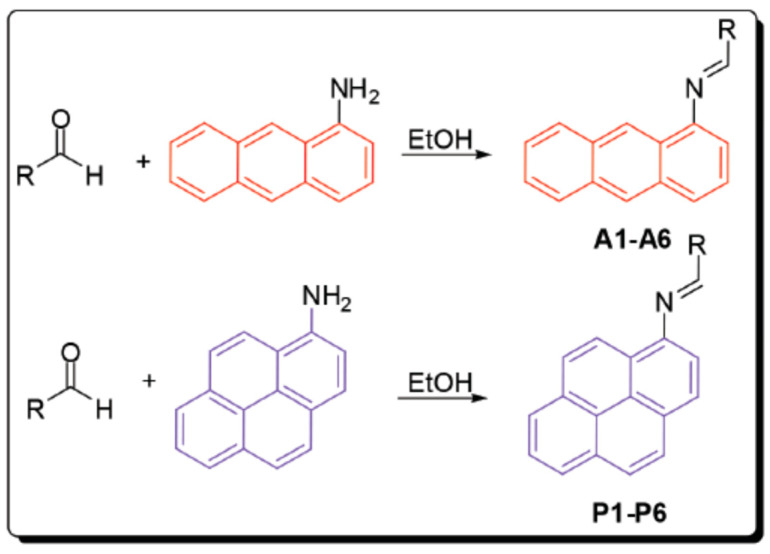
Synthesis of anthracene- and pyrene-based Schiff base derivatives [10].

Another application of anthracene-containing Schiff base was presented by Gumus et al. They designed and synthesized novel anthracene-based and pyrene-based Schiff base derivatives with a very good yield (Scheme 13). The anthracene-based derivatives were obtained via the reaction of heteroaromatic aldehydes with aminoanthracene. Many of the anthracene-based compounds showed better free radical elimination, metal chelating, reducing power, antimicrobial and DNA binding capacity compared to the pyrene-based derivatives. Three of the systems demonstrate higher antioxidant activities than all of the pyrene-containing compounds [10].

**Scheme 14:**
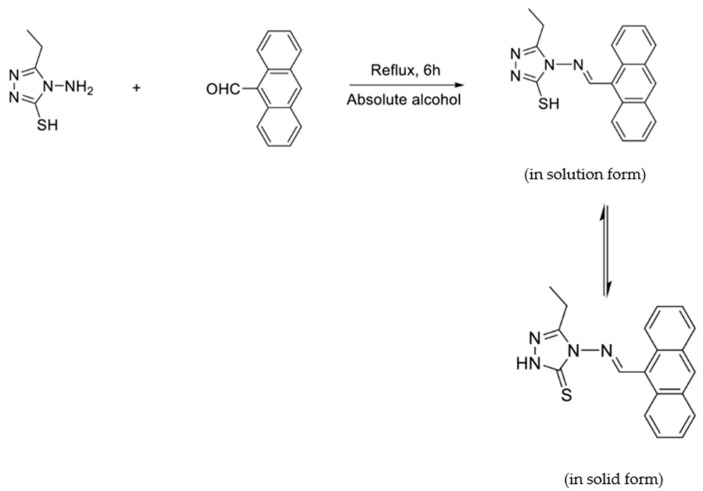
Synthesis of anthracene-based Schiff bases in solution and in solid form [70].

An example of an anthracene-containing Schiff base ligand with an increased antimicrobial activity was the 9-(((3-ethyl-5-mercapto/thio-4H-1,2,4-triazole-4-yl)imino)methyl)-anthracene synthesized by Kumari et al. through the condensation reaction of 9-anthracenecarboxaldehyde and 4-amino-3-ethyl-5-mercapto-1,2,4-triazole (Scheme 14). The spectral studies confirmed the bidentate mode of the ligand and its coordination to Cu(II), Ni(II), Cd(II) and Zn(II) metal ions. Furthermore, DPPH assay was also utilized to investigate the antioxidant potential of the complexes. The Zn(II) and Ni(II) complexes were proved to be the most active to *Pseudomonas aeruginosa* and *Candida albicans*, respectively [70].

Prakash and Ahmad also obtained and investigated new complexes of Cr(III), Ni(II) and Ti(III), with Schiff bases derived by the reaction of anthracene-9-carboxaldehyde with a number of amino acids such as L-methionine, L-glycine, L-valine, L-tryptophan and L-histidine. The results showed that the ligands were active only against Gram-positive *Staphylococcus aureus*, whereas the activity was enhanced by complexation. The ligands and the metal complexes did not exhibit anti-microbial activity on the Gram-negative *Escherichia coli*, but bacteriostatic activity of the metal complexes against *Escherichia coli* was higher than the one of the ligands [71].

**Figure 3:**
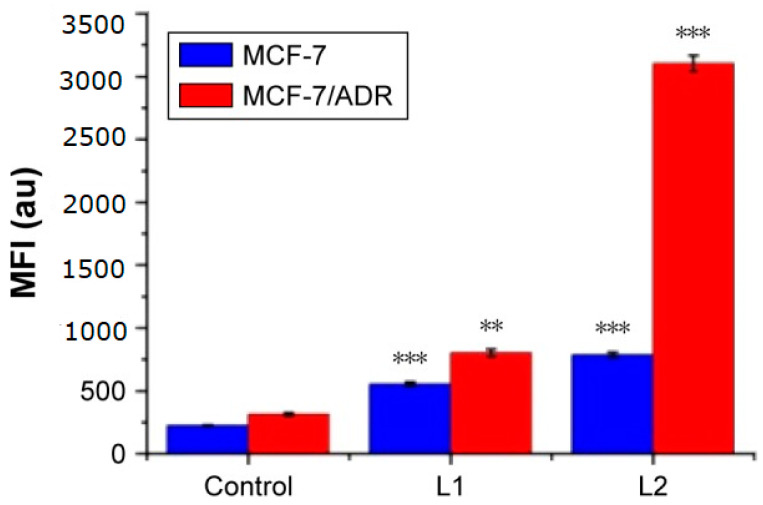
Fluorescence intensity of anthryl associated with L1 and L2 after 12 h incubation MCF-7/ADR and MCF-7 cells in 20 µM L1 and L2 [72]. ** *p* < 0.01, and *** *p* < 0.001.

Bai et al. presented another very important potential application of anthracene-based Schiff bases. They address multidrug resistance (MDR) as one of the major obstructions in cancer treatment. The authors investigated the DNA binding and cytotoxicity of two Schiff bases L1 and L2 that could be used in the fight against the extremely chemoresistant MCF-7/ADR cell with drug resistance index (DRI) 2.13. The bases induced an impairment of cell cycle progression of MCF-7/ADR and MCF-7 cell lines and suppressed cell growth (Figure 3). The results from the molecular docking and the cellular uptake implied that the tested compounds can successfully overcome P-glycoprotein efflux pump and thus exhibit cytotoxicity [72].

Another problem when it comes to the design and synthesis of anticancer drugs are the severe side effects due to toxicity, drug resistance and covalent binding to DNA. An example is the platinum-based drugs. In order to avoid these obstacles, scientists work towards the synthesis of metal-based anticancer drugs with no-covalent type of interaction with DNA. A potential metal is found to be copper. It has been already demonstrated that Cu(II) complexes possess anticancer activity. On the other hand, anthracenyl ligand moiety enhances the DNA binding tendency and, therefore, the cytotoxicity of the complexes. Although divalent copper ions are so appealing because of their decisive role in the activation of dioxygen in living organisms, at higher concentration levels, they could be damaging for some biomolecules leading to oxidative stress and neurodegenerative disorders. According to the World Health Organization (WHO), the mean daily intake of copper in adults should not surpass 12 mg for adults. On the other hand, much lower concentration levels may lead to Alzheimer’s, anemia, amyotrophic lateral sclerosis and others. Unfortunately, most of the well-known and well-used methods for determination of cupper ions are not suitable for on-line monitoring due to their low selectivity, needing expensive instruments. Fluorescent turn-on Cu^2+^ detection has become a more practical and favourable procedure in environmental chemistry and biology due to its simplicity and applications in many biological and optoelectronic systems. Anthracene-based Schiff bases have gained interest among scientists due to their selective sensor properties. Simon et al. successfully obtained a simple anthracene comprising Schiff base derivative with high selectivity and sensibility via one-pot reaction for Cu^2+^ ions detection. Their discovery has been applied in cell imaging studies with cytotoxic studies, and the compound is a potential candidate for drug delivery into the cell [73].

Another interesting field of application of anthracene-containing Schiff bases is their use as light-responsive soft actuators. Such materials are used in soft robotics and biomimetic devices. When designing light-driven soft actuators, the scientists face some challenges, such as their reliance on UV light and poor mechanical strength. Kumar et al. managed to overcome these limitations by synthesizing a functionalized anthracene dye which absorbs light in the visible region due to red-shift in the absorption band. This material was responsive to blue light. To improve its mechanical properties, the scientists mixed it in a PVA matrix. It was proven that the newly obtained Schiff bases could find applications in surgical instruments, soft robotics, biomimetic grippers, etc. [74].

Recently, the interest in fluorescent substances due to their different applications in organic light emitting devices and in the biological and materials sciences has been in-creasing. Because most organic substances are non-luminescent or weakly luminescent researchers have been working on obtaining fluorescent probes for biomolecules and optical cell imaging for the detection of diseases, such as cancer. Bovine serum albumin (BSA) is a commonly used agent for biological investigations due to its biological significand’s low cost and intrinsic fluorescence properties. On the other hand, anthracene-based compounds play the role of fluorescent probes, and, with their help, scientists can perform binding studies. These compounds find their application in the therapy of metastatic breast cancer, acute lymphoblastic leukemia, non-Hodgkin’s lymphoma and metastatic prostate cancer and are often used as chemotherapeutic agents in clinical trials. Bearing in mind all mentioned above, Densilert al. designed three anthracene-based receptors composed of amide, urea and thiourea moieties, which served as excellent fluorescent probes for BSA and optical cell imaging for cancer cells [75].

The fluorescent properties of anthracene compounds have valuable bioanalytical applications for researching the subcellular distribution and binding in healthy and cancer cells. Anthracene-containing aminophosphonates have attracted our interest in designing new antitumor therapeutics. The anthracene planar structure is important pharmacophore fragment of antineoplastic drugs. They contain essential biologically active components—an aminophosphonate group and a DNA-intercalating anthracene moiety.

In 2012, we reported the synthesis of a new anthracene-based Schiff base 9-anthrylidene-furfurylamine and three novel α-aminophosphonates bearing anthracene moiety, [N-methyl(diethoxyphosphonyl)-1-(9-anthryl)]-p-toluidine, [N-methyl (diethoxyphosphonyl)-1-(9-anthryl)]furfurylamine and [N-methyl (dimethoxyphosphonyl)-1-(9-anthryl)]-p-toluidine.

**Scheme 15:**
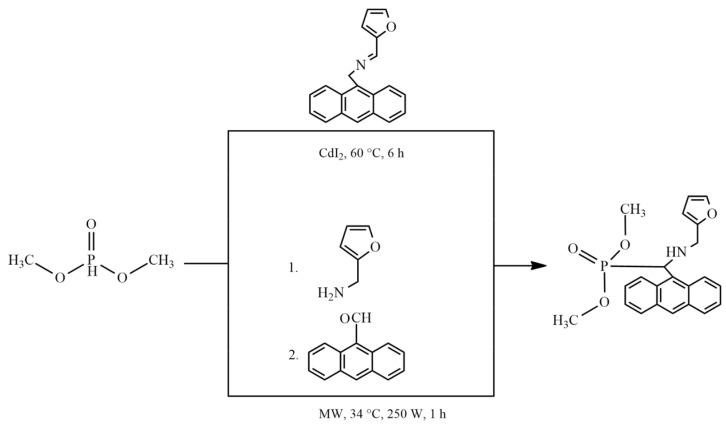
Synthetic pathways for preparation of α-aminophosphonate [N-methyl(diethoxyphosphonyl)-1-(9-anthryl)]furfurylamine [76].

Looking for efficient synthetic procedures to obtain these valuable biologically active compounds, we applied microwave synthesis as an alternative to the classical method (Scheme 15). For the first time, we received, via microwave irradiation, the Schiff base 9-anthrylidene-furfurylamine and its derivative aminophosphonate N-methyl(dimethoxyphosphonyl)-1-(9-anthryl)]furfurylamine.

We found out that microwave synthesis has a lot of benefits, as these synthetic procedures result in high yield under mild conditions and in short reaction times. We separated enantiomers of two racemic α-aminophosphonates with a chiral center and analytical and semi preparative separations were optimized. The high enantiomeric purity of the isolated enantiomers was appropriate for pharmaceutical investigations. The racemic substances and their enantiomers were investigated for cytotoxicity and genotoxicity in vivo. All investigated substances demonstrated a weak genotoxic effect. Almost half retains the proliferative capacity of the bone marrow cell population compared to the untreated control. The results allow us to assume that the hematopoietic function of the bone marrow will not be discontinued after exposure to the investigated substances [76].

### 3.1. In Vitro Antitumor Activity

These Schiff bases and their derived aminophosphonates were studied for cytotoxicity against a panel of seven cancer cell lines representative of some important species of human tumors. All substances showed dose-dependent cytotoxicity after 24 h of treatment which enabled the creation of the dose-effect curves and the calculation in accordance with the equieffective IC_50_ values shown in Table 2.

A Schiff base containing furan and anthracene rings and aminophosphonate derived from it showed twofold higher activity towards HBL 100 cells than the control drug Doxorubicin. The antiproliferative potential of these substances was comparable to that of the positive control substance used in the experiments when tested on cell lines 647-V (bladder carcinoma) and MDA-MB-231 (highly metastatic carcinoma of the breast). All tested substances demonstrated less activity than the control drug Doxorubicin after experiments with cell cultures from HeLa, HepG2 and MCF-7 cancer cell lines (Table 2).

The presence of both furan and anthracene rings is essential for the optimal activity of these compounds in the chemotherapy of malignant breast and colon disease [77].

### 3.2. In Vitro Safety Testing

The results from the validated Balb/c 3T3 (clone 31) Neutral Red Uptake Assay (3T3 NRU test) revealed dose-dependent cytotoxic activity of investigated substances. The Schiff base containing anthracene and furan rings and its corresponding aminophosphonate showed statistically significant (*p* < 0.001) cytotoxicity in a wide concentration range (1–0.07 mg/mL) to mouse embryo fibroblastic cells compared to not treated control cell cultures. However, compared to the cytotoxic effect of the positive control substance sodium dodecyl sulphate on Balb/c 3T3 cells, the cytotoxicity of the tested compounds was comparable [77].

### 3.3. Fluorescent Studies

The fluorescence activity of the anthracene-delivered agents was utilized to evaluate their intracellular distribution in HBL-100 cells following 24 h exposure to nontoxic concentrations.

The fluorescent signal of Schiff base 9-anthrylidene-p-toluidine was observed mainly in the cytoplasm of tumor cells.

**Figure 4:**
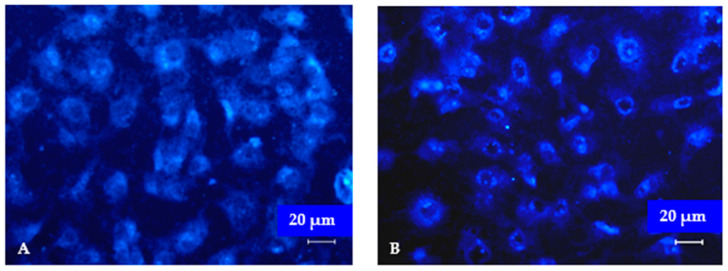
Subcellular distribution of 9-anthrylidene-p-toluidine (**A**) and the aminophosphonate [N-methyl(diethoxyphosphonyl)-1-(9-anthryl)]-p-toluidine (**B**) [77].

In contrast, the most intensive fluorescence after application of aminophosphonate [N-methyl(diethoxyphosphonyl)-1-(9-anthryl)]-p-toluidine was found in the nuclei and nuclear membranes of HBL-100 cells (Figure 4) [77].

**Scheme 16:**
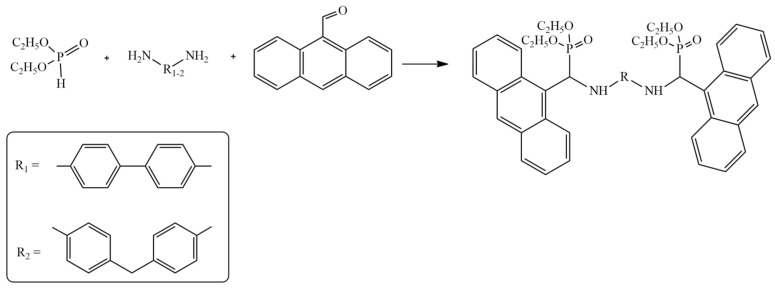
Synthesis of anthracene-containing bis(aminophosphonate)s 3 and 4 [78].

Our research continued with investigation of new bis-aminophosphonates: bis[*N*-methyl(diethoxyphosphonyl) -1-(9-anthryl)]benzidine (3) and 4.4′-bis[N-methyl(diethoxyphosphonyl)-1-(9-anthryl)]diaminodiphenylmethane (4) (Scheme 16).

### 3.4. In Vitro Antitumor Activity of Bis-Aminophosphonates

The synthesized bis-aminophosphonates 3 and 4 were studied for cytotoxicity against seven malignant human cell lines obtained from different tumors. The two aminophosphonate derivatives showed dose-dependent cytotoxicity following 24 h exposure, which enabled the construction of concentration-response curves and the calculation of the IC_50_ values. Doxorubicin was used in the comparative study as the positive control.

Both bis-aminophosphonates 3 and 4 displayed a prominent cytotoxic effect toward colon carcinoma cell line HT-29. However, comparing their effect to the other six cancer cell lines, both substances appeared to be less active than the control drug doxorubicin The IC_50_ values for the two bis-aminophosphonates were above 1 mg/mL against MDA-MB-231, HepG2 and 647-V cell lines while doxorubicin displayed an IC_50_ value less than 0.068 mg/mL. The determined values for the treatment of the other cell cultures are presented in Table 3. In all studied cancer cell lines, no activity augmentation was observed after 48 h treatment, a possible explanation is the high level of P-glycoprotein, particularly in HT-29 cells.

### 3.5. In Vitro Safety Testing of Bis-Aminophosphonates

Dose-dependent cytotoxic activity of bis-aminophosphonates 3 and 4 after exposure for 24 h was found from the validated Balb/c 3T3 (clone 31) Neutral Red Uptake Assay (3T3 NRU test). The investigated compounds showed noticeable cytotoxicity (*p* < 0.05 and *p* < 0.001) at concentrations equal or higher than 0.464 mg/mL compared to untreated control cell cultures. The compounds 3 and 4 were toxic to 50% of the cells at concentrations of 0.545 ± 0.018 and 0.510 ± 0.019 mg/mL, respectively. These values compared to the IC_50_ value of 0.0562 ± 0.0003 mg/mL obtained for SDS evidenced that the cytotoxicity of both tested substances was approximately 10-fold lower. Moreover, no considerable cytotoxic activity was noted after a 48 h treatment with concentrations of up to 1 mg/mL.

The performed experiments revealed that the investigated bis-aminophosphonates 3 and 4 are substances with a very low toxicity to no tumorigenic rodent embryo cells and displayed an adequate xenobiotic clearance after prolonged exposure [78].

### 3.6. In Vivo Safety Testing of Bis-Aminophosphonates

The antiproliferative and clastogenic potential of the two anthracene-containing bis-aminophosphonates 3 and 4 were investigated in vivo in laboratory rodents. The results are presented in Table 4. It was proved that the investigated substances at a dose of 10 mg/kg evoked only a moderate damage on chromosomes structure. The portion of metaphases with aberrant chromosomes was found to be 5.00% for 3 and 5.50 for 4 after 24 h treatment. These values were markedly lower (*p* < 0.001) than those for the drug Mitomycin C with known genotoxic effect which was used in the experiments as a positive referent compound.

The results presented in Table 4 also show that the number of aberrant metaphases in the experimental groups injected with a dose of 100 mg/kg of 3 and 4, was greater than the number of metaphases observed in the experimental groups treated with 10 mg/kg of each bis-aminophosphonate. The highest percentage of cells with aberrations, i.e., 11.5 ± 0.5% was obtained when the experimental group was tested with 4 at a dose of 100 mg/kg. However, this value was approximately three times lower (*p* < 0.001) than the percentage of damaged metaphases (30.5 ± 2.36%) after Mitomycin C treatment. In addition, the values of metaphases with aberrations lowered after the application of both compounds at a dose of 100 mg/kg for 48 h compared to the values determined after 24 h treatment (Table 4).

For all tested ICR animals, chromatid fragmentation and centromere-centromeric fusions were present in the bone marrow cells. Statistical evaluation of the data concerning the mitotic indices showed that both substances under investigation demonstrated rather lower antiproliferative effects as compared to the reference Mitomycin C (Table 4). Slight difference in the inhibition of cell proliferation was detected between the groups treated with 100 mg/kg and 10 mg/kg of the tested bis-aminophosphonates 3 and 4. Compound 3 injected at 10 mg/kg dose inhibited cell division by about 40%.

The data from the in vivo and in vitro experiments indicated that both of the tested bis-aminophosphonatess had moderate clastogenic and low cytotoxicity to healthy cells. These results motivated us to perform studies on the fluorescent properties of the compounds to visualize their cellular uptake and intracellular distribution [78].

### 3.7. Fluorescent Studies of Bis-Aminophosphonates

The fluorescence activity of the anthracene-containing compounds 3 and 4 were implemented in the evaluation of their cellular compartmentalization in Balb/c 3T3 (clone 31) and HT-29 cells after treatment with sub-IC_50_ dose. The results showed predominant nuclear localization in mouse embryo 3T3 cells. Faint, diffuse fluorescence of the cytoplasm was also evident. Contrarily, in HT-29 cells the bis-aminophosphonates 3 and 4 were mainly localized in the nuclear membranes and late S-phase associated nuclear structures. The obtained fluorescent images confirmed the in vitro cytotoxic activities of the tested bis-aminophosphonates [78].

### 3.8. Polyaminophosphonates

The development of new therapeutic approaches provoked the creation of effective phosphorus-containing polymeric systems for treating different diseases, including cancer, with enormous possibilities for advanced pharmaceutical science. The macromolecular technique offers the opportunity of improving the therapy of various human pathologies and overcoming problems associated with drug side effects and the length of drug action [78,79,80,81,82,83].

Among the variety of macromolecular structures designed for drug conjugation, the polymers with phosphoester (C-O-P-O-C) repeating units in the backbone occupy a particularly important place because they can degrade into non-toxic and biocompatible fragments under physiological conditions. These polymers can possess reactive functional groups in the main or in the side chain, which allows for the conjugation of bioactive molecules and thus providing the construction of novel drug delivery systems with augmented therapeutic indexes [84,85,86,87,88].

To the best of our knowledge, we were the first who received poly(oxyethylene aminophosphonete)s change off copolymers built only of poly(ethylene glycol) and aminophosphonate units derived from poly(oxyethylene H-phosphonate)s [89]. The addition of Schiff Bases to biodegradable polymers like poly(oxyethylene H-phosphonate)s looks like a feasible procedure in the synthesis of new polymer drug carriers, as well as of new polymers displaying own biological activity.

**Scheme 17:**
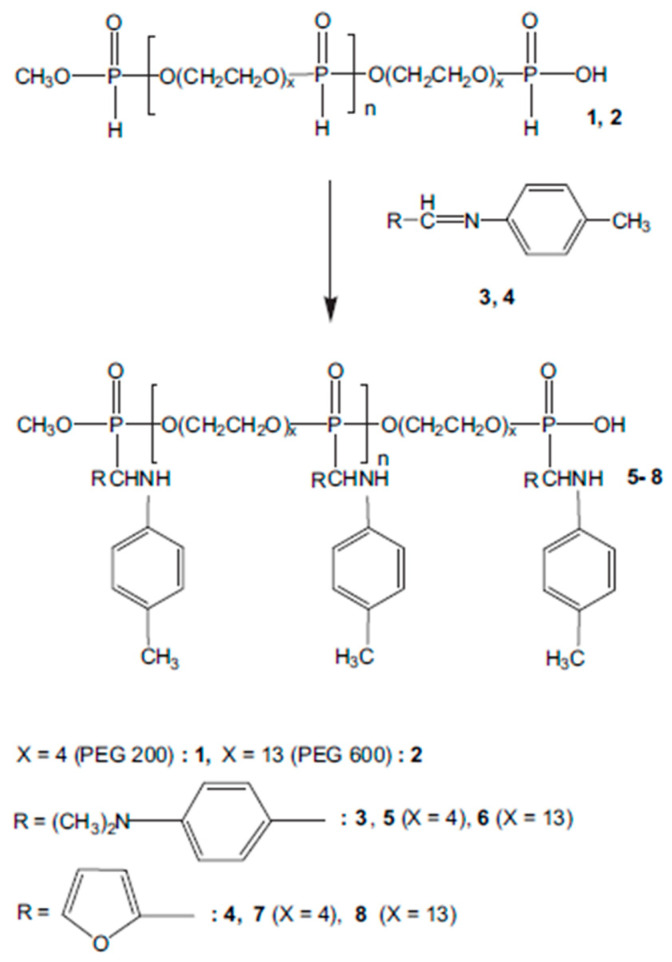
Synthesis of poly(oxyethylene aminophosphonate)s 5–8 [90].

We implemented the developed procedure for the preparation of poly(oxyethylene aminophosphonate)s (5–8) via the addition of two Schiff bases, N-(4-dimethylaminobenzylidene)-p-toluidine (3) and N-furfurylidene p-toluidine (4), to poly(oxyethylene H-phosphonate)s (Scheme 17) [90]. The latter were synthesized using PEGs with different molar masses.

**Scheme 18:**
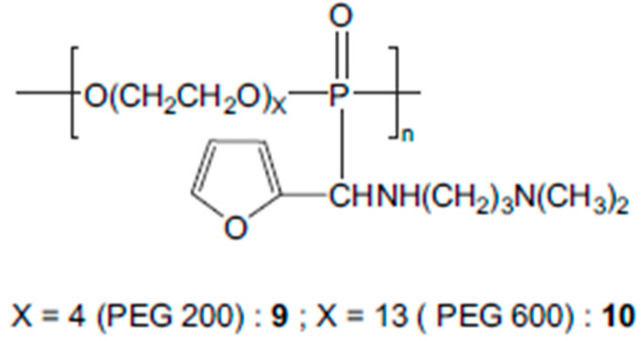
Poly(oxyethylene aminophosphonate)s 9 and 10 repeating units [90].

The poly(aminophosphonate)s 9 and 10 were obtained in similar manner from a Schiff base N,N-dimethyl-N’-furfurylidene-1,3-diaminopropane and poly(oxyethylene H-phosphonate)s derived from PEG200 and PEG600 (Scheme 18).

The poly(oxyethylene aminophosphonate)s 5–10 have coordination centers in their repeating units and are candidates as new biodegradable polymer carriers for the physical conjugation of bioactive compounds. The polymers were evaluated for cytotoxicity in four malignant cell lines following 72 h treatment at varying concentrations (Table 5). Polymers 6, 7, 8 and 10 showed strong antiproliferative effects and low micromolar IC_50_ values, whereas polymer 9 was less active, and 5 exhibited only marginal toxicity.

The 2-furyl-p-toluidine moiety and the longer (13 units) PEG segments abundant in **8** were identified as structural prerequisites affording superior activity. The analogues obtained from the Schiff bases N-(4-dimethylaminobenzylidene)-p-toluidine and N,N-dimethyl-N’-furfurylidene-1,3-diaminopropane displayed lower activity than 8. Despite the Schiff base residue, however, in all subseries of poly(aminophosphonate)s, the decrease in the length of the PEG segments (from 13 oxyethylene units in PEG600 to 4 units in PEG200) evoke a noticeable reduction in relative potency, most prominent in the case of 5 where almost total loss of activity was observed. The cytotoxicity of polymers 6, 7, 8 and 10 was found to be comparable to the reference drug cisplatin, i.e., findings that gave us reason to consider them as a novel class of aminophosphonate-delivered cytotoxic substances [89].

Poly(alkylene H-phosphonate)s are one of the most promising drug carriers because they contain highly reactive P–H groups in the repeating fragments, which can bond covalently with low molecular weight drugs in mild reaction conditions. The presence of highly polar repeating P–O groups enables physical immobilization of low molecular agents. They are capable of generating new biologically active substances, like aminophosphonate fragments, connected to their polymer chains. This method makes it possible to receive a new type of polymer prodrugs and new polymer drug carriers.

Introducing aminophosphonate fragments bearing DNA intercalating anthracene rings to the biodegradable polymer will contribute to the development of novel cytotoxic agents with upgraded characteristics.

The anthracene-based substances exhibit a broad spectrum of anticancer activity and play a significant role as chemotherapeutic drugs in cancer therapy, act as tubulin polymerization inhibitors and find useful bioanalytical applications as chromophores for fluorescence measurements [91,92,93,94].

In search of new cytotoxic molecules, we have developed a synthesis of new polyphosphoesters containing anthracene-based aminophosphonate fragments, poly(oxyethylene aminophosphonate)s and poly[oxyethylene (aminophosphonate-co-H-phosphonate)]s.

In the copolymers, the content of the hydrophilic H-phosphonate units is greater than the aminophosphonate fragments, which makes them soluble in water. The fluorescence spectra of all polymers revealed the emission maxima in the blue—blue-green spectral range.

The copolymers were evaluated for in vitro cytotoxicity on a panel of seven human epithelial cancer cell lines.

These new compounds appeared to belong to the low-toxicity group of DNA intercalators. Investigated polymers appear promising for the development of active antineoplastic drugs for chemotherapy of malignant breast and liver disease.

The results of the fluorescent microscopy evaluation well paralleled the results of in vitro cytotoxicity and revealed apoptotic and necrotic alterations in breast tumor cells and hepatocellular carcinoma cells after prolonged exposure, which gives insights on the potential mechanism of cancer cell eradication. The clastogenic effects of the elaborated polymers have been reduced over time. Although, the tested polymers showed only slight suppression on bone marrow cell division, as compared to the referent drug (Mitomycin C), they significantly inhibit the mitotic processes compared to the untreated cells [95].

**Scheme 19:**
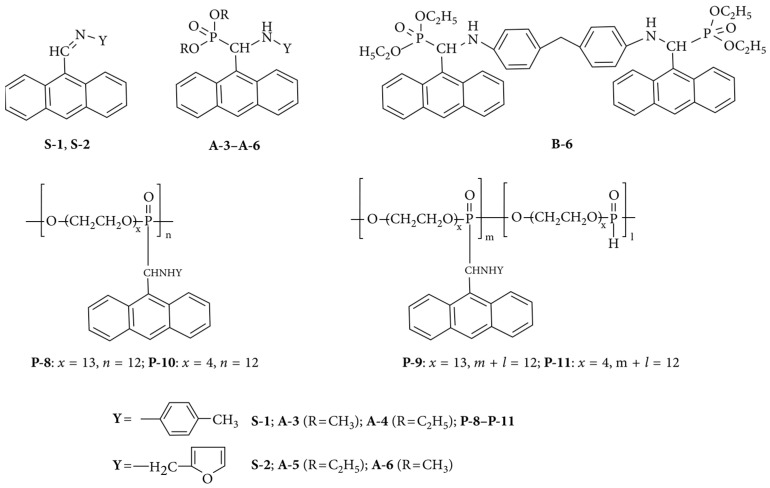
Structure of anthracene-derived Schiff bases S-1 and anthracene and furan-containing S-2, *α*-aminophosphonates A-3–A-6, bis-aminophosphonate B-6, and polyphosphoesters P-8–P-11 [96].

In summary, we have studied the methods of obtaining the anthracene-derived Schiff base S-1 and anthracene and furan-containing Schiff base S-2, α-aminophosphonates A-3–A-6, bis-aminophosphonate B-6 (R, S diastereomer), and polyphosphoesters P-8–P-11, bearing aminophosphonate fragments (Scheme 19).

Anthracene-delivered Schiff base S-1 and the corresponding α-aminophosphonates A-3 and A-4 and the polyphosphoesters P-8–P-11, and anthracene and furan-bearing Schiff base S-2 and its derivatives, the substances A-5 and A-6, two enantiomers A-5a and A-5b were investigated for antiproliferative activity against human leukemic cell lines, exploiting cisplatin as a control anticancer drug.

Among the investigated substances A-3, A-4 and A-5, aminophosphonate A-5 with anthracene and furan rings in the structure, displayed the highest inhibitory activity against all studied human epithelial cancer cell lines. This result motivated us to introduce aminophosphonate building blocks with anthracene and furan residues into the polyphosphoester backbone to yield novel polyaminophosphonates P-12 and P-13. They possess hydrolytically unstable phosphoester linkages in their backbones. The polymers P-8 and P-10 are built only of aminophosphonate repeating fragments, while the copolymers P-9 and P-11–P-13 are composed of polyoxyethyleneaminophosphonate blocks and hydrophilic polyoxyethylene H-phosphonate segments. The antiproliferative potential of the new macromolecules was tested against a series of human cancer cell lines: K-562, SKW-3, REH and HL-60 (Table 6).

The results indicate that aminophosphonate A-4 and polymers P-8 and P-9 could be developed as promising new candidates for cytotoxic agents [96].

## 4. Conclusions

Schiff Bases and their derivatives as aminophosphonates and poly(aminophosphonate)s are capable of inhibiting tumor cell growth and inducing cell death in different test systems, necessitating further exploration for the delineation of their antineoplastic potential. Furthermore, the presence of anthracene and furan-containing moieties impart additional valuable properties such as fluorescent activity and enhanced penetration via biological membranes beneficial in the design of novel pharmaceutical agents. Polyphosphoesters are appropriate macromolecules for developing drug delivery systems as they are biodegradable in a physiological medium and yield biocompatible and non-toxic degradation products. The availability of pharmacophore fragments and a biodegradable and non-toxic polymer backbone leads to obtaining macromolecular drug carriers with their own pharmacological activity and opens an investigational avenue for the future exploration thereof.

## Figures and Tables

**Table 1:** Comparative cytotoxic activity of furyl and N-tolyl containing Schiff base and the corresponding aminophosphonate [62].

Cell Line	IC_50_ Value (µM) ^a^					
	Cisplatin	2	3	4	5	6
LAMA-84 ^b^	18.2 ± 1.7	39.9 ± 2.1	251.9 ± 7.3	>400.0	71.2 ± 2.4	119.4 ± 6.3
K-562 ^b^	25.7 ± 2.1	29.9 ± 1.9	212.9 ± 12.1	352.9 ± 11.7	22.9 ± 0.9	42.4 ± 3.0
HL-60 ^b^	7.8 ± 1.1	>400.0	>400.0	163.4 ± 5.3	74.8 ± 2.7	>400.0
HL-60/DOX ^c,d^	14.5 ± 1.4	68.6 ± 4.0	226.1 ± 5.9	190.0 ± 4.7	115.2 ± 7.1	107.2 ± 4.1

^a^ Arithmetic mean_standard deviation of at least 6 independent experiments. ^b^ Chronic myeloid leukemia. ^c^ Acute promyelocyte leukemia. ^d^ Multi-drug-resistant (mdr) phenotype due to overexpression of MRP-1 (ABCC1) transporter.

**Table 2:** Cytotoxic activity of substances 1–5 against control drug Doxorubicin in a panel of seven human tumor cell lines after 24 h course (MTT assay) [77].

Cell Lines	IC_50_ Value (µM) ^a^					
	Doxorubicin	1	2	3	4	5
MCF-7	<125	1590 ± 60.1	912.3 ± 49.1	1111.1 ± 59.3	>4600	236.4 ± 7.1
MDA-MB-231	<125	424 ± 24.7	1578.9 ± 498.2	2172.8 ± 56.8	>4600	165.5 ± 2.4
HBL-100	257.6 ± 20.23	1095 ± 95.4	1614 ± 63.2	49.4 ± 14.8	1685.9 ± 53.11	165.5 ± 2.4
HepG2	<125	636 ± 38.9	421 ± 17.5	2049 ± 61.7	>4600	260 ± 4.7
HT-29	1067.1 ± 23.9	282.7 ± 10.6	701.8 ± 42.1	>4600	>4600	260 ± 2.4
647-V	<125	459.4 ± 14.1	280.7 ± 7	1481.5 ± 51.9	>4600	165.5 ± 2.4
138.6HeLa	<125	600.7 ± 14.1	1228 ± 0.03	>4600	4480.4 ± 138.6	283.7 ± 2.4

^a^ Values are means ± standard deviation from three consecutive experiments.

**Table 3:** Cell growth inhibitory of compounds 3 and 4 against referent drug Doxorubicin in a panel of human tumor cell lines after 24 h treatment (MTT-dye reduction assay) [78].

Cell Lines	IC_50_ Value (µM) ^a^		
	Doxorubicin	3	4
MCF-7	<125.1	865.8 ± 46.8	757.6 ± 13.3
MDA-MB-231	<125.1	>1200	>1176
HBL-100	257.8 ± 20.2	>1200	1129.4 ± 28.5
HepG2	<125.1	>1200	>1176
HT-29	1067.1 ± 23.9	539.1 ± 5.2	512.9 ± 5.2
647-V	<125.1	>1200	>1176
HeLa	<125.1	1103.1 ± 6.8	1034.1 ± 6.2

^a^ Values are means ± standard error of mean from three consecutive experiments.

**Table 4:** Proliferative activity and clastogenic effect of ICR mice bone marrow cells after i.p. application of the bis-aminophosphonates 3 and 4 [78].

Bis- Aminophosphonates and Doses	Time after Treatment	Number of Metaphases Scored	Type of Chromosome Aberrations					Mitotic Index (‰) (X ± m)	Percentage of Cells with Aberrations (X ± m)
			Breaks	Fragments	Rearrangements				
					c/c	t/t	c/t		
(**3**) 10 mg/kg	24 h	400	0	3	17	0	0	12.07 ± 0.99	5.00 ± 0.84
	48 h	450	4	5	11	0	0	11.18 ± 0.68	4.37 ± 0.59
(**3**) 100 mg/kg	24 h	400	10	13	13	3	0	8.20 ± 0.72	9.75 ± 0.88
	48 h	400	9	7	8	1	0	7.27 ± 0.83	6.25 ± 0.70
(**4**) 10 mg/kg	24 h	400	5	5	10	2	0	8.39 ± 0.35	5.50 ± 0.33
	48 h	400	3	8	6	1	0	8.93 ± 0.51	4.50 ± 0.33
(**4**) 100 mg/kg	24 h	400	21	8	17	0	0	10.08 ± 0.73	11.50 ± 0.50
	48 h	400	8	8	13	0	0	7.90 ± 0.34	7.25 ± 0.53
Mit. C 3.5 mg/kg	24 h	200	17	30	7	1	0	5.49 ± 0.19	30.50 ± 2.36
	48 h	400	17	24	20	0	0	7.29 ± 0.34	15.80 ± 0.81
DMSO	24 h	500	1	1	4	0	0	15.14 ± 0.46	1.40 ± 0.30
	48 h	500	2	0	2	0	0	12.47 ± 1.07	0.80 ± 0.32
Control 0.9% NaCl	24 h	700	4	0	4	0	0	20.06 ± 1.38	1.14 ± 0.34
	48 h	500	0	0	3	0	0	16.88 ± 0.56	0.60 ± 0.30

**Table 5:** Cytotoxicity of the poly(oxyethylene aminophosphonate)s 5–10 against reference drug cisplatin, evaluated by MTT-dye reduction assay after 72 h continuous exposure [90].

Compounds	IC_50_ (µmol/L)			
	K-562 ^a^	LAMA-84 ^a^	HL-60/Dox ^a,^ *	HL-60 ^b^
**5**	>400	>400.0	>400.0	105.9
**6**	14.9	17.2	27.2	19.2
**7**	15.2	14.0	20.4	19.9
**8**	13.7	12.3	14.4	14.2
**9**	62.3	88.8	159.1	157.5
**10**	18.3	41.9	16.2	66.2
Cisplatin	25.7	18.2	14.5	7.8

^a^ Chronic myeloid leukemia. ^b^ Acute promyelocyte leukemia; (* multi-drug resistant sub-line).

**Table 6:** In vitro cytotoxicity against a panel of human tumor cell lines: K-562, SKW-3, REH and HL-60 the referent drug cisplatin [96].

Compounds	IC_50_ (µM)			
	K-562 (CML)	SKW-3 (CLL)	REH (ALL)	HL-60 (APML)
**S-1**	80.8 ± 11.4	57.2 ± 4.6	19.8 ± 2.7	47.7 ± 6.9
**S-2**	89.7 ± 9.1	34.3 ± 7.1	18.3 ± 3.2	37.3 ± 4.6
**A-3**	>200	37.0 ± 5.6	30.5 ± 7.8	35.6 ± 6.1
**A-4**	**18.7 ± 4.9**	**8.4 ± 1.5**	**14.1 ± 1.9**	**21.2 ± 4.4**
**A-5**	139.1 ± 8.1	76.3 ± 7.9	114 ± 14.7	76.8 ± 12.9
**A-6**	47.3 ± 6.5	51.4 ± 7.3	42.8 ± 6.9	64.1 ± 9.8
**A-5a**	58.6 ± 2.0	10.5 ± 2.5	21.4 ± 0.7	30.2 ± 2.1
**A-5b**	79.7 ± 3.3	20.5 ± 2.0	17.6 ± 0.8	30.3 ± 2.1
**B6**	>200	187.2 ± 14.4	165.9 ± 21.5	139.1 ± 11.3
**P-8**	**35.6 ± 7.3**	**3.1 ± 1.2**	**7.9 ± 1.4**	**2.1 ± 0.6**
**P-9**	**24.8 ± 1.1**	**5.4 ± 1.2**	**16.8 ± 1.2**	**5.5 ± 1.2**
**P-10**	163.0 ± 22.3	38.5 ± 4.3	98.7 ± 8.8	49.4 ± 7.3
**P-11**	>200	61.1 ± 9.2	113.4 ± 10.5	116.9 ± 10.4
**P-12**	26.9 ± 5.9	29.5 ± 5.2	33.8 ± 6.4	40.1 ± 7.6
**Cisplatin**	**18.6 ± 3.9**	**11.2 ± 0.8**	**17.2 ± 2.1**	**8.1 ± 1.7**

ALL-acute lymphoid leukemia; CML-chronic myelocytic leukemia; APML-acute promyeloid leukemia; CLL-chronic lymphocytic leukemia.

## Data Availability

Not applicable.
